# Riemann Boundary Value Problem for Triharmonic Equation in Higher Space

**DOI:** 10.1155/2014/415052

**Published:** 2014-07-08

**Authors:** Longfei Gu

**Affiliations:** Department of Mathematics, Linyi University, Linyi, Shandong 276000, China

## Abstract

We mainly deal with the boundary value problem for triharmonic function with value in a universal Clifford algebra: Δ^3^[*u*](*x*) = 0, *x* ∈ *R* ^*n*^∖∂Ω, *u* ^+^(*x*) = *u* ^−^(*x*)*G*(*x*) + *g*(*x*), *x* ∈ ∂Ω, (*D* ^*j*^ *u*)^+^(*x*) = (*D* ^*j*^ *u*)^−^(*x*)*A*_*j*_ + *f*_*j*_(*x*), *x* ∈ ∂Ω, *u*(∞) = 0, where (*j* = 1,…, 5)  ∂Ω is a Lyapunov surface in *R* ^*n*^, *D* = ∑_*k*=1_^*n*^ *e*_*k*_(∂/∂*x*_*k*_) is the Dirac operator, and *u*(*x*) = ∑_*A*_ *e*_*A*_ *u*_*A*_(*x*) are unknown functions with values in a universal Clifford algebra Cl(*V*_*n*,*n*_). Under some hypotheses, it is proved that the boundary value problem has a unique solution.

## 1. Introduction and Preliminaries

The theory of Riemann boundary value problems in complex plane has been systematically developed in [1, 2]. It is an interesting topic to generalize the classical Riemann boundary value problems theory to Clifford analysis. In [3–6], and so forth, many interesting results about boundary value problem and Riemann Hilbert problems for monogenic functions in Clifford analysis are presented. In [7], Green's function for the Dirichlet problem for polyharmonic equations was studied. The aim of this paper is to study the Riemann boundary value problem for triharmonic functions. At first, based on the higher order Cauchy integral representation formulas in [8, 9] and the Plemelj formula, we give some properties of triharmonic functions in Clifford analysis, for example, the mean value theorem, the Painlevé theorem, and so forth. Furthermore, on the basis of the above results, we consider the following Riemann boundary value problems:

(1) Δ3[u](x)=0, x∈Rn∖∂Ω,u+(x)=u−(x)A+g(x), x∈∂Ω,(Dju)+(x)=(Dju)−(x)Aj+fj(x), x∈∂Ω,|u(∞)|≤C∗,

(2) Δ3[u](x)=0, x∈Rn∖∂Ω,u+(x)=u−(x)G(x)+g(x), x∈∂Ω,(Dju)+(x)=(Dju)−(x)Aj+fj(x), x∈∂Ω,u(∞)=0,

where (*j* = 1,…, 5).

In (1) and (2), *A* and *A*_*j*_ are invertible constants; we denote the inverse elements as *A* ^−1^ and *A*_*j*_^−1^.  *u*(*x*), (*D* ^*j*^ *u*)(*x*), *g*(*x*), *f*_*j*_(*x*) ∈ *H* ^*β*^(∂Ω, Cl(*V*_*n*,*n*_)), *j* = 1,…, 5, 0 < *β* ≤ 1. The explicit solutions for (1) are given and the boundary value problem (2) is shown to have a unique solution under some hypotheses.

Let *V*_*n*,*s*_ (0 ≤ *s* ≤ *n*) be an *n*-dimensional (*n* ≥ 1) real linear space with basis {*e*_1_, *e*_2_,…, *e*_*n*_}, and Cl(*V*_*n*,*s*_) the universal Clifford algebra over *V*_*n*,*s*_. For more information on Cl(*V*_*n*,*s*_) (0 ≤ *s* ≤ *n*), we refer to [10–12].

Throughout this paper, suppose Ω is an open, bounded nonempty subset of *R* ^*n*^ with a Lyapunov boundary ∂Ω, denoting Ω^+^ = Ω, $\Omega^{-}=R^{n}\setminus \bar{\Omega }$ . In this paper, for simplicity, we will only consider the case of *s* = *n*. The operator *D* is given as

(3) $$\begin{array}{c} D=\overset{n}{\underset{k=1}{\sum }}e_{k}\frac{\partial }{\partial x_{k}}:C^{r}\left(\Omega ,\text{Cl}\left(V_{n,n}\right)\right)⟶C^{r-1}\left(\Omega ,\text{Cl}\left(V_{n,n}\right)\right). \end{array}$$

Let *u* be a function with value in Cl(*V*_*n*,*n*_), defined in Ω, and the operator *D* acts on the function *u* from the left and from the right, which is being governed by the following rule:

(4) $$\begin{array}{c} D\left[u\right]=\overset{n}{\underset{k=1}{\sum }}\underset{A}{\sum }e_{k}e_{A}\frac{\partial u_{A}}{\partial x_{k}},\\ \left[u\right]D=\overset{n}{\underset{k=1}{\sum }}\underset{A}{\sum }e_{A}e_{k}\frac{\partial u_{A}}{\partial x_{k}}. \end{array}$$

**Definition 1 .**

A compact surface Γ is called Lyapunov surface with Hölder exponent *α*, if the following conditions are satisfied.

(i) At each point *x* ∈ Γ there is a tangential space.
(ii) There exists a number *r*, such that for any point *x* ∈ Γ the set Γ∩*B*_*r*_(*x*) (Lyapunov ball) is connected and parallel lines to the outer normal *α*(*x*) intersect at not more than one point.
(iii) The normal *α*(*x*) is Hölder continuous on Γ; that is, there are constants *C* > 0 and 0 < *α* ≤ 1 such that for *x*, *y* ∈ Γ
(5) $$\begin{array}{c} \left|\alpha \left(x\right)-\alpha \left(y\right)\right|\leq C{\left|x-y\right|}^{\alpha }. \end{array}$$

Let Ω be an open nonempty subset of *R* ^*n*^ with a Lyapunov boundary, *u*(*x*) = ∑_*A*_ *e*_*A*_ *u*_*A*_(*x*), where *u*_*A*_(*x*) are real functions; *u*(*x*) is called a Hölder continuous function on $\bar{\Omega }$ if the following condition is satisfied:

(6) |u(x1)−u(x2)| =[∑A|uA(x1)−uA(x2)|2]1/2≤C|x1−x2|α,

where for any $x_{1},x_{2}\in \bar{\Omega }$ , *x*_1_ ≠ *x*_2_, 0 < *α* ≤ 1, *C* is a positive constant independent of *x*_1_, *x*_2_.

Let *H* ^*α*^(∂Ω, Cl(*V*_*n*,*n*_)) denote the set of Hölder continuous functions with values in Cl(*V*_*n*,*n*_) on ∂Ω (the Hölder exponent is *α*, 0 < *α* < 1). We denote the norm in *H* ^*α*^(∂Ω, Cl(*V*_*n*,*n*_)) as

(7) $$\begin{array}{c} {||u||}_{\left(\alpha ,\partial \Omega \right)}={||u||}_{\infty }+{||u||}_{\alpha }, \end{array}$$

where

(8)  ||u||∞∶=sup⁡x∈∂Ω⁡|u(x)|, ||u||α∶=sup⁡x1,x2∈∂Ωx1≠x2|u(x1)−u(x2)||x1−x2|α.

**Lemma 2 .**

The Hölder space *H* ^*α*^(∂Ω, *Cl*(*V*_*n*,*n*_)) is a Banach space with norm (7).

Denote the fundamental solutions of *D* ^*j*^ (*j* = 1,2,…, 6) by

(9) H1(x)=1ωnxρn(x),H2(x)=12−n1ωn1ρn−2(x),H3(x)=12(2−n)1ωnxρn−2(x),H4(x)=12(2−n)(4−n)1ωn1ρn−4(x),H5(x)=18(2−n)(4−n)1ωnxρn−4(x),H6(x)=18(2−n)(4−n)(6−n)1ωn1ρn−6(x),

where *ρ*(*x*) = (∑_*i*=1_^*n*^ *x*_*i*_^2^)^1/2^ and *ω*_*n*_ denotes the area of the unit sphere in *R* ^*n*^ (*n* ≥ 3, *n* ≠ 4, *n* ≠ 6).

We will introduce the following operators:

(10) $$\begin{array}{c} \left(F_{\partial \Omega }u\right)\left(x\right)=\int_{\partial \Omega }2H_{1}\left(y-x\right)d\sigma_{y}u\left(y\right),\;x\in R^{n}\setminus \partial \Omega , \end{array}$$

(11) $$\begin{array}{c} \left(S_{\partial \Omega }u\right)\left(x\right)=\int_{\partial \Omega }2H_{1}\left(y-x\right)d\sigma_{y}u\left(y\right),\;x\in \partial \Omega , \end{array}$$

where *u* ∈ *H* ^*α*^(∂Ω, Cl(*V*_*n*,*n*_)).

**Lemma 3 (see [5]).**

The integral operator *S*_∂Ω_ in (11) is a bounded linear operator mapping the function space *H* ^*α*^(∂Ω, *Cl*(*V*_*n*,*n*_)) into itself; that is, there exists a positive constant *M* such that, for all *u* ∈ *H* ^*α*^(∂Ω; *Cl*(*V*_*n*,*n*_)),

(12) $$\begin{array}{c} {||S_{\partial \Omega }u||}_{\left(\alpha ,\partial \Omega \right)}\leq M{||u||}_{\left(\alpha ,Cl(V_{n,n}\right)}. \end{array}$$

## 2. Some Properties for Triharmonic Functions

**Theorem 4 (Gauss-mean value formula for triharmonic functions, see [6, 13]).**

Suppose Δ^3^[*u*] = 0 in *R* ^*n*^; then, for any *x* ∈ *R* ^*n*^,

(13) u(x)=1ωnRn−1∫∂B(x,R)u(y)dS −R22nΔ[u](x)−R48n(n+2)Δ2[u](x),

or

(14) u(x)=nωnRn∫B(x,R)u(y)dV−R22(n+2)Δ[u](x)−R48(n+2)(n+4)Δ2[u](x),

where *ω*_*n*_ denotes the area of the unit sphere in *R* ^*n*^.

**Corollary 5 .**

Suppose Δ^3^[*u*] = 0 in *R* ^*n*^ and |*u*(*x*)| = *O*(|*x*|) (|*x* | →*∞*); then Δ[*u*] = 0 and Δ^2^[*u*] = 0 in *R* ^*n*^.

**Corollary 6 .**

Suppose Δ^3^[*u*] = 0 in *R* ^*n*^ and *u*(*x*) is bounded in *R* ^*n*^; then *u*(*x*) ≡ *C*.

**Theorem 7 .**

Suppose Δ^3^[*u*] = 0 in *R* ^*n*^∖∂Ω, and for *x* ∈ ∂Ω, *u* ∈ *C* ^6^(*R* ^*n*^∖∂Ω, *Cl*(*V*_*n*,*n*_)), [*u*]^+^(*x*) = [*u*]^−^(*x*) ∈ *C* ^5^(∂Ω, *Cl*(*V*_*n*,*n*_)), and, moreover, *D* ^*j*^[*u*]^+^(*x*) = *D* ^*j*^[*u*]^−^(*x*) ∈ *H* ^*α*_*j*_^(∂Ω, *Cl*(*V*_*n*,*n*_)), 0 < *α*_*j*_ ≤ 1, where *j* = 0,1,…, 5. Then Δ^3^[*u*] = 0 in *R* ^*n*^.

**Theorem 8 .**

Let $u\in C^{6}(\Omega^{-},Cl(V_{n,n}))\cap C^{5}(\bar{\Omega^{-}},Cl(V_{n,n}))$ , Δ^3^[*u*] = 0 in Ω^−^, *D* ^*j*^[*u*](*x*) ∈ *H* ^*α*_*j*_^(∂Ω, *Cl*(*V*_*n*,*n*_)), 0 < *α*_*j*_ ≤ 1 (*j* = 0,1,…, 5), and |*u*(*x*)| = *O*(1) (|*x*| → *∞*); then for *x* ∈ Ω^−^

(15) $$\begin{array}{c} u\left(x\right)=\overset{5}{\underset{j=0}{\sum }}{\left(-1\right)}^{j+1}\int_{\partial \Omega }H_{j+1}\left(y-x\right)d\sigma_{y}D^{j}\left[u\right]\left(y\right)+C, \end{array}$$

where *H*_*j*_(*y* − *x*) is as in (9) and *C* is a constant.

**Proof**

For *y* ∈ ∂Ω, denote *D* ^*j*^[*u*](*y*) = −Ψ_*j*_(*y*), (*j* = 0,1,…, 5). For *x* ∈ *R* ^*n*^∖∂Ω, denoting

(16) $$\begin{array}{c} \Theta \left(x\right)=\overset{5}{\underset{j=0}{\sum }}{\left(-1\right)}^{j}\int_{\partial \Omega }H_{j+1}\left(y-x\right)d\sigma_{y}\Psi_{j}\left(y\right),\\ u^{*}\left(x\right)=\{\begin{array}{cc} -\Theta \left(x\right), & x\in \Omega^{+}\\ u\left(x\right)-\Theta \left(x\right), & x\in \Omega^{-}, \end{array} \end{array}$$

then Δ^3^[u*] = 0 in *R* ^*n*^∖∂Ω. By using Plemelj formula, combining with weak singularity of *H*_*j*_(*x*) (*j* ≥ 2), we obtain the following:

(17) $$\begin{array}{c} D^{j}{\left[u^{*}\right]}^{+}\left(x\right)=D^{j}{\left[u^{*}\right]}^{-}\left(x\right)\in H^{\alpha_{j}^{\prime }}\left(\partial \Omega ,\text{Cl}\left(V_{n,n}\right)\right), \end{array}$$

where 0 < *α*_*j*_′ ≤ 1 (*j* = 0,1,…, 5). By using Theorem 7, we have Δ^3^[u*] = 0 in *R* ^*n*^. It is clear that we have |u*(*x*)| = *O*(1) (|*x* | →*∞*). In view of Corollary 6, then the results follow.

**Corollary 9 .**

Let $u\in C^{6}(\Omega^{-},Cl(V_{n,n}))\cap C^{5}\left(\bar{\Omega^{-}},Cl\left(V_{n,n}\right)\right)$ , Δ^3^[*u*] = 0 in Ω^−^, *D* ^*j*^[*u*](*x*) ∈ *H* ^*α*_*j*_^(∂Ω, *Cl*(*V*_*n*,*n*_)), 0 < *α*_*j*_ ≤ 1 (*j* = 0,1,…, 5), and |*u*(*x*)| = *O*(1) (|*x* | →*∞*), then

(18) $$\begin{array}{c} D^{j}\left[u\right]\left(\infty \right)=0,\;j=1,\ldots ,5, \end{array}$$

**Remark 10 .**

When the condition |*u*(*x*)| = *O*(1) (|*x* | →*∞*) in Corollary 9 is replaced by *u*(*∞*) = 0, then the results in Corollary 9 are still valid.

## 3. Riemann Boundary Value Problem for Triharmonic Functions

In this section, we will consider the Riemann boundary value problem (1); the explicit expression of the solution is given.

**Theorem 11 .**

The Riemann boundary value problem (1) is solvable and the solution can be written as

(19) $$\begin{array}{c} u\left(x\right)=\{\begin{array}{cc} \overset{6}{\underset{i=1}{\sum }}\Psi_{i}\left(x\right)+C, & x\in \Omega ,\\ \overset{6}{\underset{i=1}{\sum }}\Psi_{i}\left(x\right)+CA^{-1}, & x\in \Omega^{-}, \end{array} \end{array}$$

where

(20) $$\begin{array}{c} \Psi_{1}\left(x\right)=\{\begin{array}{cc} -\int_{\partial \Omega }H_{6}\left(y-x\right)d\sigma_{y}f_{5}\left(y\right), & x\in \Omega^{+}\\ -\int_{\partial \Omega }H_{6}\left(y-x\right)d\sigma_{y}f_{5}\left(y\right)A_{5}^{-1}, & x\in \Omega^{-} \end{array} \end{array}$$

(21) $$\begin{array}{c} \Psi_{2}\left(x\right)=\{\begin{array}{cc} \int_{\partial \Omega }H_{5}\left(y-x\right)d\sigma_{y}{\tilde{f}}_{4}\left(y\right), & x\in \Omega^{+}\\ \int_{\partial \Omega }H_{5}\left(y-x\right)d\sigma_{y}{\tilde{f}}_{4}\left(y\right)A_{4}^{-1}, & x\in \Omega^{-} \end{array} \end{array}$$

(22) $$\begin{array}{c} \Psi_{3}\left(x\right)=\{\begin{array}{cc} -\int_{\partial \Omega }H_{4}\left(y-x\right)d\sigma_{y}{\tilde{f}}_{3}\left(y\right), & x\in \Omega^{+}\\ -\int_{\partial \Omega }H_{4}\left(y-x\right)d\sigma_{y}{\tilde{f}}_{3}\left(y\right)A_{3}^{-1}, & x\in \Omega^{-} \end{array} \end{array}$$

(23) $$\begin{array}{c} \Psi_{4}\left(x\right)=\{\begin{array}{cc} \int_{\partial \Omega }H_{3}\left(y-x\right)d\sigma_{y}{\tilde{f}}_{2}\left(y\right), & x\in \Omega^{+}\\ \int_{\partial \Omega }H_{3}\left(y-x\right)d\sigma_{y}{\tilde{f}}_{2}\left(y\right)A_{2}^{-1}, & x\in \Omega^{-} \end{array} \end{array}$$

(24) $$\begin{array}{c} \Psi_{5}\left(x\right)=\{\begin{array}{cc} -\int_{\partial \Omega }H_{2}\left(y-x\right)d\sigma_{y}{\tilde{f}}_{1}\left(y\right), & x\in \Omega^{+}\\ -\int_{\partial \Omega }H_{2}\left(y-x\right)d\sigma_{y}{\tilde{f}}_{1}\left(y\right)A_{1}^{-1}, & x\in \Omega^{-} \end{array} \end{array}$$

(25) $$\begin{array}{c} \Psi_{6}\left(x\right)=\{\begin{array}{cc} \int_{\partial \Omega }H_{1}\left(y-x\right)d\sigma_{y}\tilde{g}\left(y\right), & x\in \Omega^{+}\\ \int_{\partial \Omega }H_{1}\left(y-x\right)d\sigma_{y}\tilde{g}\left(y\right)A^{-1}, & x\in \Omega^{-} \end{array} \end{array}$$

(26) f~4(x)=f4(x)−∫∂ΩH2(y−x)dσyf5(y)(−1+A5−1A4),x∈∂Ω,

(27) f~3(x)=f3(x)+∫∂ΩH3(y−x)dσyf5(y)(−1+A5−1A3)−∫∂ΩH2(y−x)dσyf~4(y)(−1+A4−1A3), x∈∂Ω,

(28) f~2(x)=f2(x)−∫∂ΩH4(y−x)dσyf5(y)(−1+A5−1A2)+∫∂ΩH3(y−x)dσyf~4(y)(−1+A4−1A2)−∫∂ΩH2(y−x)dσyf~3(y)(−1+A3−1A2), x∈∂Ω,

(29) f~1(x)=f1(x)+∫∂ΩH5(y−x)dσyf5(y)(−1+A5−1A1)−∫∂ΩH4(y−x)dσyf~4(y)(−1+A4−1A1)+∫∂ΩH3(y−x)dσyf~3(y)(−1+A3−1A1)−∫∂ΩH2(y−x)dσyf~2(y)(−1+A2−1A1), x∈∂Ω,

(30) g~(x)=g(x)−∫∂ΩH6(y−x)dσyf5(y)(−1+A5−1A)+∫∂ΩH5(y−x)dσyf~4(y)(−1+A4−1A)−∫∂ΩH4(y−x)dσyf~3(y)(−1+A3−1A)+∫∂ΩH3(y−x)dσyf~2(y)(−1+A2−1A)−∫∂ΩH2(y−x)dσyf~1(y)(−1+A1−1A),x∈∂Ω.

## 4. Existence of Solutions for Riemann Boundary Value Problem for Triharmonic Functions

**Theorem 12 .**

Suppose *G*(*x*) ∈ *H* ^*α*^(∂Ω, *Cl*(*V*_*n*,*n*_))  0 < *α* < 1 and *G*(*x*) satisfies the following condition:

(31) $$\begin{array}{c} 2^{n-2}{||1-G\left(x\right)||}_{\left(\alpha ,\partial \Omega \right)}\left(M+1\right)<1, \end{array}$$

where *M* is the positive constant mentioned in Lemma 3. Then (2) admits a unique solution.

**Proof**

Denoting *w*(*x*) = *D* ^5^[*u*](*x*) then *w* ^+^(*x*) = *w* ^−^(*x*)*A*_5_ + *f*_5_(*x*), *x* ∈ ∂Ω. Moreover, by *D* ^5^[*u*](*∞*) = 0. we get the following:

(32) $$\begin{array}{c} \omega \left(x\right)=\{\begin{array}{cc} \int_{\partial \Omega }H_{1}\left(y-x\right)d\sigma_{y}f_{5}\left(y\right), & x\in \Omega^{+}\\ \int_{\partial \Omega }H_{1}\left(y-x\right)d\sigma_{y}f_{5}\left(y\right)A_{5}^{-1}, & x\in \Omega^{-}, \end{array} \end{array}$$

With Ψ_1_(*x*) being as in (20), it is easy to check that

(33) $$\begin{array}{c} D^{5}\left(u-\Psi_{1}\right)\left(x\right)=0,\;x\in R^{n}\setminus \partial \Omega . \end{array}$$

Denoting Δ^2^ *u*(*x*) − Δ^2^Ψ_1_(*x*)∶ = *φ*_1_(*x*), *x* ∈ *R* ^*n*^∖∂Ω, and using (Δ^2^ *u*)^+^(*x*) = (Δ^2^ *u*)^−^(*x*)*A*_4_ + *f*_4_(*x*), *x* ∈ ∂Ω, we conclude that

(34) $$\begin{array}{c} \varphi_{1}^{+}\left(x\right)=\varphi_{1}^{-}\left(x\right)A_{4}+{\tilde{f}}_{4}\left(x\right),\;x\in \partial \Omega , \end{array}$$

where ${\tilde{f}}_{4}(x)\in H^{\tilde{\beta }}(\partial \Omega ,\text{Cl}(V_{n,n}))$ , $0<\tilde{\beta }\leq 1$ being as in (26). By Corollary 9, it is clear that *φ*_1_(*∞*) = 0, and we then get the following representation formula:

(35) $$\begin{array}{c} \varphi_{1}\left(x\right)=\{\begin{array}{cc} \int_{\partial \Omega }H_{1}\left(y-x\right)d\sigma_{y}{\tilde{f}}_{4}\left(y\right), & x\in \Omega^{+}\\ \int_{\partial \Omega }H_{1}\left(y-x\right)d\sigma_{y}{\tilde{f}}_{4}\left(y\right)A_{4}^{-1}, & x\in \Omega^{-}. \end{array} \end{array}$$

Analogously, we find with Ψ_2_(*x*) from (21) that Δ^2^ *u*(*x*) − Δ^2^Ψ_1_(*x*) − Δ^2^Ψ_2_(*x*) = 0, *x* ∈ *R* ^*n*^∖∂Ω. Denote

(36) D3u(x)−D3Ψ1(x)−D3Ψ2(x)∶=φ2(x),x∈Rn∖∂Ω.

Using the condition (*D* ^3^ *u*)^+^(*x*) = (*D* ^3^ *u*)^−^(*x*)*A*_3_ + *f*_3_(*x*), *x* ∈ ∂Ω, we obtain that

(37) $$\begin{array}{c} \varphi_{2}^{+}\left(x\right)=\varphi_{2}^{-}\left(x\right)A_{3}+{\tilde{f}}_{3}\left(x\right),\;x\in \partial \Omega , \end{array}$$

where ${\tilde{f}}_{3}(x)\in H^{\tilde{\beta }}(\partial \Omega ,\text{Cl}(V_{n,n}))$ , $0<\tilde{\beta }\leq 1$ being as in (27). By Corollary 9, it is clear that *φ*_2_(*∞*) = 0, and we then get the following representation formula:

(38) $$\begin{array}{c} \varphi_{2}\left(x\right)=\{\begin{array}{cc} \int_{\partial \Omega }H_{1}\left(y-x\right)d\sigma_{y}{\tilde{f}}_{3}\left(y\right), & x\in \Omega^{+}\\ \int_{\partial \Omega }H_{1}\left(y-x\right)d\sigma_{y}{\tilde{f}}_{3}\left(y\right)A_{3}^{-1}, & x\in \Omega^{-}. \end{array} \end{array}$$

Here Ψ_3_(*x*) being as in (22), then we have *D* ^3^ *u*(*x*) − *D* ^3^Ψ_1_(*x*) − *D* ^3^Ψ_2_(*x*) − *D* ^3^Ψ_3_(*x*) = 0, *x* ∈ *R* ^*n*^∖∂Ω. We denote

(39) $$\begin{array}{c} \Delta u\left(x\right)-\Delta \Psi_{1}\left(x\right)-\Delta \Psi_{2}\left(x\right)-\Delta \Psi_{3}\left(x\right)∶=\varphi_{3}\left(x\right) \end{array}$$

and use the condition (Δ*u*)^+^(*x*) = (Δ*u*)^−^(*x*)*A*_2_ + *f*_2_(*x*), *x* ∈ ∂Ω. We conclude that

(40) $$\begin{array}{c} \varphi_{3}^{+}\left(x\right)=\varphi_{3}^{-}\left(x\right)A_{2}+{\tilde{f}}_{2}\left(x\right),\;x\in \partial \Omega , \end{array}$$

where ${\tilde{f}}_{2}(x)\in H^{\tilde{\beta }}(\partial \Omega ,\text{Cl}(V_{n,n}))$ , $0<\tilde{\beta }\leq 1$ being as in (28). Corollary 9 ensures that *φ*_3_(*∞*) = 0; then

(41) $$\begin{array}{c} \varphi_{3}\left(x\right)=\{\begin{array}{cc} \int_{\partial \Omega }H_{1}\left(y-x\right)d\sigma_{y}{\tilde{f}}_{2}\left(y\right), & x\in \Omega^{+}\\ \int_{\partial \Omega }H_{1}\left(y-x\right)d\sigma_{y}{\tilde{f}}_{2}\left(y\right)A_{2}^{-1}, & x\in \Omega^{-}. \end{array} \end{array}$$

Use the same way again, Ψ_4_(*x*) being as in (23), that Δ*u*(*x*) − ΔΨ_1_(*x*) − ΔΨ_2_(*x*) − ΔΨ_3_(*x*) − ΔΨ_4_(*x*) = 0, *x* ∈ *R* ^*n*^∖∂Ω. Denoting

(42) $$\begin{array}{c} Du\left(x\right)-\overset{4}{\underset{i=1}{\sum }}D\Psi_{i}\left(x\right)∶=\varphi_{4}\left(x\right),\;x\in R^{n}\setminus \partial \Omega \end{array}$$

and using (*Du*)^+^(*x*) = (*Du*)^−^(*x*)*A*_1_ + *f*_1_(*x*), *x* ∈ ∂Ω, we get

(43) $$\begin{array}{c} \varphi_{4}^{+}\left(x\right)=\varphi_{4}^{-}\left(x\right)A_{1}+{\tilde{f}}_{1}\left(x\right),\;x\in \partial \Omega , \end{array}$$

where ${\tilde{f}}_{1}(x)\in H^{\tilde{\beta }}(\partial \Omega ,\text{Cl}(V_{n,n}))$ , $0<\tilde{\beta }\leq 1$ being taken from (29). By Corollary 9, it is clear that *φ*_4_(*∞*) = 0, and we then get the following representation formula:

(44) $$\begin{array}{c} \varphi_{4}\left(x\right)=\{\begin{array}{cc} \int_{\partial \Omega }H_{1}\left(y-x\right)d\sigma_{y}{\tilde{f}}_{1}\left(y\right), & x\in \Omega^{+}\\ \int_{\partial \Omega }H_{1}\left(y-x\right)d\sigma_{y}{\tilde{f}}_{1}\left(y\right)A_{1}^{-1}, & x\in \Omega^{-}. \end{array} \end{array}$$

Finally, we use Ψ_5_(*x*) as defined in (24) and get *Du*(*x*) − ∑_*i*=1_^4^ *D*Ψ_*i*_(*x*) = 0, *x* ∈ *R* ^*n*^∖∂Ω. Define

(45) $$\begin{array}{c} u\left(x\right)-\overset{5}{\underset{i=1}{\sum }}\Psi_{i}\left(x\right)∶=\varphi_{5}\left(x\right),\;x\in R^{n}\setminus \partial \Omega . \end{array}$$

Working with the condition *u* ^+^(*x*) = *u* ^−^(*x*)*G*(*x*) + *g*(*x*) we arrive at

(46) $$\begin{array}{c} \varphi_{5}^{+}\left(x\right)=\varphi_{5}^{-}\left(x\right)G\left(x\right)+\tilde{g}\left(x\right),\;x\in \partial \Omega , \end{array}$$

where $\tilde{g}(x)\in H^{\tilde{\beta }}(\partial \Omega ,\text{Cl}(V_{n,n}))$ , $0<\tilde{\beta }\leq 1$ being taken from (30). It is clear that *φ*_5_(*∞*) = 0. We obtain that

(47) Dφ5=0, Rn∖∂Ωφ5+(x)=φ5−(x)G(x)+g~(x), x∈∂Ωφ5(∞)=0.

We only need to consider the existence of solutions to (47). The solution to this problem may be written in the form

(48) $$\begin{array}{c} \varphi_{5}\left(x\right)=\int_{\partial \Omega }H_{1}\left(y-x\right)d\sigma_{y}\varphi_{6}\left(y\right), \end{array}$$

where *φ*_6_(*y*) is a Hölder continuous function to be determined on ∂Ω. Then, by using Plemelj formula, (47) can be reduced to an equivalent singular integral equation for *φ*_6_,

(49) φ6(x)=[φ6(x)2−∫∂ΩH1(y−x)dσyφ6(y)](1−G(x))+g~(x),x∈∂Ω.

Letting *T* denote the integral operator defined by the right hand side of (49), we get

(50) $$\begin{array}{c} \left(T\varphi_{6}\right)\left(x\right)=\left[\varphi_{6}\left(x\right)-\left(S_{\partial \Omega }\varphi_{6}\right)\left(x\right)\right]\frac{\left(1-G\left(x\right)\right)}{2}+\tilde{g\left(x\right)}. \end{array}$$

For any *ω*_1_, *ω*_2_ ∈ *H* ^*α*^(∂Ω, Cl(*V*_*n*,*n*_)), we have

(51) ||Tω1−Tω2||(α,∂Ω) ≤2n−12||ω1−ω2||(α,∂Ω)||1−G||(α,∂Ω)(1+M).

Under the condition (31), the integral operator *T* is a contraction operator mapping the Banach space *H* ^*α*^(∂Ω, Cl(*V*_*n*,*n*_)) into itself, which has a unique fixed point for the operator *T*. Thus, there exists a unique solution to (47). The proof is done.
